# Waterbird response to variable-timing of drawdown in rice fields after winter-flooding

**DOI:** 10.1371/journal.pone.0204800

**Published:** 2018-10-04

**Authors:** Kristin A. Sesser, Monica Iglecia, Matthew E. Reiter, Khara M. Strum, Catherine M. Hickey, Rodd Kelsey, Daniel A. Skalos

**Affiliations:** 1 Point Blue Conservation Science, Petaluma, California, United States of America; 2 Audubon California, Sacramento, California, United States of America; Consejo Superior de Investigaciones Cientificas, SPAIN

## Abstract

Wetland loss and degradation have been extensive across the world, especially in California’s Central Valley where over 90% of the natural wetlands have been converted to agricultural and urban uses. In the Central Valley today, a much smaller network of managed wetlands and flooded agricultural fields supports almost five million waterfowl and half a million shorebirds. Over 50% of waterbird habitat in the Central Valley is provided by flooded agricultural land, primarily rice (*Oryza sativa*). Each year non-breeding waterbird habitat decreases in the late winter as flooded agricultural fields are drained after waterfowl hunting season in late-January to prepare for the next crop. This study evaluated a practice called ‘variable drawdown’ that involves delaying the removal of water from rice fields by 1, 2, and 3 weeks to extend the availability of flooded habitat later into February and March. We studied waterbird response to variable drawdown in 2012 and 2013 at twenty rice farms throughout the northern half of the Central Valley. The staggered drawdown created a mosaic of water depths throughout the six-week study period. The 3-week delay in drawdown supported more dabbling ducks than earlier drawdowns in the first half of the study and more shorebirds and long-legged wading birds during the second half of the study. The timing of highest use of each drawdown treatment differed for each waterbird guild; dabbling ducks, geese and swans benefited at the beginning, then long-legged wading birds, followed by shorebirds. Despite the presence of appropriate water depths for shorebirds across the treatments during the entire study period, shorebird densities were highest near the end of the study when the 3-week-delayed drawdown was providing the majority of the habitat on the landscape. This suggests that shorebirds may have concentrated in our study fields due to decreasing availability of shallow water habitat elsewhere. The practice of variable drawdown successfully extended the availability of waterbird habitat provided by post-harvest flooded rice fields later into winter.

## Introduction

Wetland loss and degradation has been extensive across the world [1,2] and the Central Valley of California, USA is no exception where over 90% of the estimated two million hectares of natural wetlands have been converted to agricultural and urban uses [3,4]. Now, a much smaller network of managed wetlands and flooded agricultural fields fills the role of historic natural wetlands [5]. Despite this loss of wetland habitat, nearly three million ducks, two million geese, and 500,000 shorebirds continue to migrate through or overwinter in this region [6,7], making the Central Valley an internationally important area for migratory waterbirds in the Pacific Flyway [8,9].

In California, over 50% of potential waterbird habitat is on agricultural land (over 1 million acres in the Central Valley) [9,10]. In the northern half of the Central Valley, the Sacramento Valley, rice (*Oryza sativa*) fields flooded after harvest to decompose rice residue provide approximately 70% of the flooded habitat in some winters [9], supporting over 50 species of waterbirds [11–13]. During November, December, and January waterbirds in the Sacramento Valley exploit resources provided by a combination of winter-flooded rice fields as well as publicly- and privately-owned wetlands managed year-round, primarily, for waterfowl conservation. The removal of water from flooded rice fields (‘drawdown’) typically occurs from late-January to mid-February; soon after the close of the waterfowl hunting season. The drawdown of rice leaves managed wetlands to support the majority of the waterbirds in the Sacramento Valley in March and April. Precipitation may extend flooding of rice into March [14,15], however rainfall is highly variable from year to year (30-year precipitation [cm]: February minimum = 0.2, maximum = 30.7, mean = 8.2; March minimum = 0, maximum = 20.1, mean = 6.0). This creates a challenge for waterbirds since large numbers are still using the Sacramento Valley; waterfowl generally begin their northbound migration out of the Sacramento Valley in early March [9] and many shorebird species are still wintering in March as peak migration out of and through the region is in April [7]. Shorebirds presumably take advantage of the short period of shallow water created from the traditional drawdown practice in rice, but then must move to other habitats, such as managed wetlands, or leave the Sacramento Valley [16,17].

Habitat and food resource availability are critical for energy accumulation in migrating waterbirds [18,19] and we identified the time period immediately after winter drawdown of rice as an important opportunity to extend habitat availability in winter-flooded rice fields to support migratory waterbirds [9,20,21]. We collaborated with rice growers and the rice industry, represented by the California Rice Commission to develop a practice to stagger drawdown of rice fields to promote a diversity of water depths over time. A diversity of water depths is critical to providing habitat for a diversity of waterbirds [22–24]. Shorebirds, which represent the smallest body size of individual waterbirds likely to use rice, require water depths less than 15 cm [12,22,25]. Dabbling ducks most often use deeper depths, ranging from 10–30 cm in rice fields [12,22]. Geese and swans and long-legged wading birds have less restrictive water depth requirements, using both non-flooded and flooded habitats [12,22,26].

Our objective for this study was to assess the ability of a staggered (‘variable’) drawdown practice to create habitat for multiple waterbird guilds later in the winter as measured by the waterbird response. We predicted that fields with the most delayed drawdown would benefit dabbling ducks and geese and some wading birds initially, when deeper water would still be present, and as drawdown continued, the shallower water would benefit shorebirds.

## Methods

### Study area

**Fig 1:**
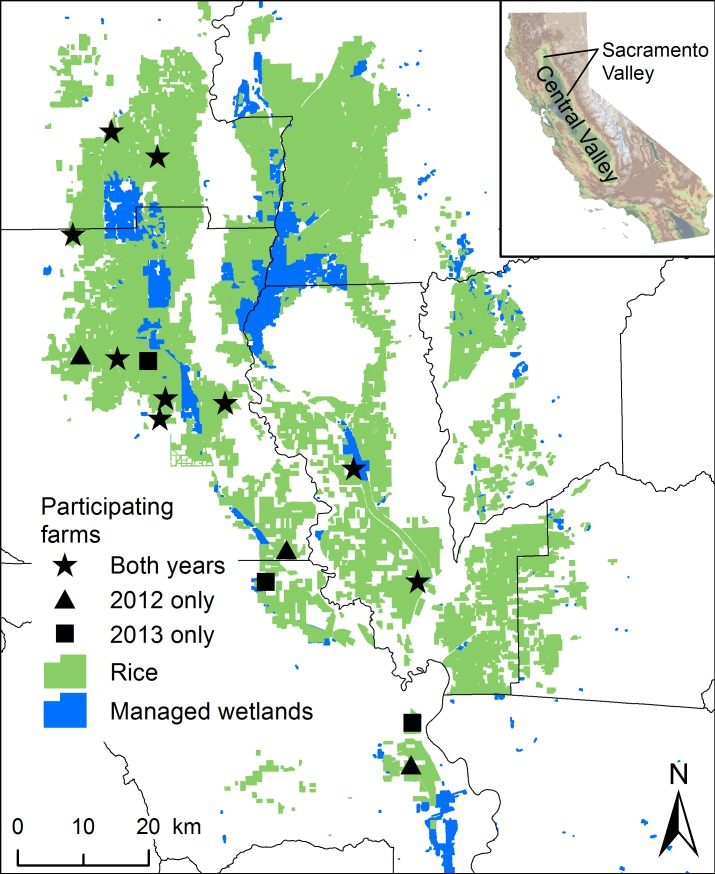
Map of study area. Location of 15 rice farms (stars = both years, triangle = 2012, square = 2013) where waterbird surveys were conducted in the context of rice (green) and managed wetlands (blue) on the landscape in the Sacramento Valley, California.

We conducted this study in the Sacramento Valley which is the northern portion of the Central Valley of California, USA (39°12’N, 122°00’W; Fig 1). This area is characterized by warm, dry summers and mild, wet winters; average annual rainfall is 51 cm. In the Sacramento Valley, there are 35,122 ha of managed wetlands which complement the approximately 215,000 ha of rice grown annually [20], and during winter, approximately 107,000 ha of rice are flooded post-harvest for residue decomposition and to provide waterbird habitat [27].

Most Sacramento Valley rice fields are conventionally grown (100% of fields in our study) in a rice mono-culture on laser-leveled fields resulting in relatively even water depths across entire fields when flooded. Fields are mechanically prepared for planting in April and May and seeded in May. Fields are flooded through most of the growing season and then dried out in preparation for harvest in August and September. Post-harvest practices can vary, but growers generally use machinery to put residual rice residue in contact with the soil to enhance decomposition during the wet season which begins in October. Common methods include chopping the residue into smaller pieces, disking, and stomping (smashing residue into water). State regulations enacted in the 1990s restricted the amount of post-harvest rice stubble that could be burned (Rice Straw Burning Act, 1991) and resulted in an increase in the amount of rice that is intentionally flooded in winter to promote decomposition of rice residue. The shift from burning to flooding for residue decomposition had the unintended consequence of creating high value habitat for waterbirds [13,28,29] while providing agronomic benefits to farmers [30] and increasing the recreational value of rice through waterfowl hunting opportunities. In our study, each field had only one combination of the post-harvest practices described above, and within farms, fields were treated in very similar ways; thus there was more variation in post-harvest practices among farms than within them. All participating fields were flooded continuously for 2.5–4 months prior to the beginning of drawdown. This resulted in a uniform appearance of the field substrate during drawdown. The action of the water over the winter reduces many of the differences among fields resulting from differences in post-harvest practices such as micro-topography and the amount of rice residue.

### Variable drawdown

We developed a water management strategy designed to extend the availability of shallow water on the landscape after traditionally drawn down fields became dry by staggering the timing of drawdown of water in multiple rice fields over four weeks (hereafter variable drawdown). Variable drawdown was implemented using three fields at each farm. An additional field at each farm was also included in the study that was drained according to the traditional timeline used by growers (no delay, ND), with the boards in the water control structures removed after the end of waterfowl hunting season (30 January 2012 and 4 February 2013). Drawdown of the ‘variable drawdown’ fields was staggered over the next three consecutive weeks: one field had drawdown delayed by 1 week (1WD), another by 2 weeks (2WD), and another by 3 weeks (3WD). Growers were instructed to have a minimum starting depth of ten centimeters, but no other instructions were given other than the drawdown dates. The variable drawdown practice is part of a federal conservation incentive (payment for ecosystem services) program run by the U.S. Department of Agriculture’s Natural Resources Conservation Service; six of the fifteen farms in our study were enrolled in this program (five of these for both years).

### Study design

We studied the waterbird response (differences in abundance) to variable drawdown at 12 rice farms in 2012 and 11 farms in 2013; eight farms were the same in both years (Fig 1). Farm selection was opportunistic, depending on the willingness of growers to participate in the study, but provided reasonable spatial representation of the landscape. Participating fields were selected by the grower, and treatment (ND, 1WD, 2WD, 3WD) was assigned at random with the exception of the ND treatment at five farms, where growers thought the fields would dry too slowly if placed in the later treatments. Within farms, rice fields differed in size (N = 65; minimum = 13.0 ha; maximum = 105.1 ha; mean = 45.3 ha) and shape and were divided into a varying number of subunits called paddies that are separated by internal earthen levees. We selected 3–6 paddies to survey within fields of each treatment at each farm using Generalized Random Tessellation Stratified sampling methodology, which enabled the selection of spatially balanced random locations with respect to treatment [31]. Each paddy contained only one survey location and we considered the field to be the sample unit. The amount of flooded habitat in the surrounding landscape is known to affect waterbird densities [32,33] so we included all treatments in clusters at all farms so the distribution of habitat in the surrounding landscape should be represented similarly in all treatments on a farm and thus across the study. Farms were separated by 0.6–19 km.

### Data collection

We conducted waterbird surveys during daylight hours (07:50–17:59) twice per week for six weeks from 30 January to 9 March 2012 and 4 February to 15 March 2013 (Table 1) for a total of 12 visits per survey location per year. In 2013, two growers began preparing their fields for planting before we finished the last survey, resulting in only 11 visits to five fields; however these fields were already dry. We conducted surveys from the edge of each selected rice paddy and used a 200-m fixed-radius or the internal levees separating paddies (whichever was closer) to define the survey area [22]. This kept the probability of detection high (~1) for waterbirds; though we have no reason to suspect that the probability of detection varied across treatments, so even if <1, it would not influence comparisons. The survey areas ranged in size from 0.8–5.4 ha depending on how wide the paddies were, with an average of 2.2 ha. Where possible, we varied the order in which we visited survey areas during daylight hours to avoid bias in counts due to the effects of time of day. Observers identified all waterbirds to species and counted all individuals using the survey area, but did not include birds that only flew over. Each survey area was scanned for at least two minutes (range 2–28 min, median = 3 min). Surveys were not conducted in inclement weather, i.e. winds ≥ 40 kph, heavy fog, or rain.

**Table 1:** Drawdown dates and sample sizes for the four treatments in each year of the study.

Treatment	2012				2013			
	Drawdown date	Total fields	Total paddies	Total surveys	Drawdown date	Total fields	Total paddies	Total surveys
ND	Jan 30	12	63	704	Feb 4	10	49	567
1WD	Feb 6	12	62	689	Feb 11	11	55	649
2WD	Feb 13	12	69	770	Feb 18	11	60	708
3WD	Feb 20	11	64	712	Feb 25	11	61	720
Total:		47	258	2875		43	225	2644

ND, no delay; 1WD, 1-week delay; 2WD, 2-week delay; 3WD, 3-week delay.

During each waterbird survey, we recorded water depth to the nearest 2.5 cm in each survey area using a stake placed in the center of the paddy at 200 m from the survey point. We also visually estimated the percent of the survey area that was flooded (standing water), saturated (moisture clearly visible in soil), or dry (no moisture visible in soil).

### Data analysis

We included four focal waterbird guilds in our analysis that are commonly found in rice fields and are priorities for regional conservation planning by the Central Valley Joint Venture [9]: dabbling ducks (Anseriformes), geese and swans (Anseriformes), long-legged wading birds, hereafter ‘wading birds’ (Pelicaniformes and Gruiformes), and shorebirds (Charadriiformes).

We modeled waterbird counts for each of the four focal waterbird guilds using generalized linear mixed modeling. We included random effects for each farm and individual field to account for potential correlations in our nested data [34]. For each waterbird guild, our response variable was the sum of all the counts within a field (2–6 survey areas per field) for each survey occasion. This removed spatial autocorrelation due to surveys of paddies within the same field on the same day and also reduced zero-inflation in the data. To account for variation in the size of survey area, we included an offset term in each model that was equal to the natural logarithm of the total area (ha) surveyed in the field [34]. We calculated the area (ha) surveyed for waterbirds at each survey area using ArcMap Version 10.2.1 [35]. The start date was offset by five calendar days between the two years of the study (Jan 30 in 2012 and Feb 4 in 2013) so when we used date in analyses, it was based on the start of the study (days 1–40), not the calendar date.

Despite pooling data from all paddies in a field, the variance to mean ratio of our bird counts was greater than one, suggesting they were overdispersed. Thus for shorebirds and wading birds, we used the negative binomial distribution and applied the glmer.nb function from the lme4 package in R [34,36,37]. For dabbling ducks and geese and swans, models using the negative binomial distribution would not converge, thus, we used the binomial distribution and applied the glmer function from the lme4 package in R to model dabbling ducks and geese and swans as the probability of occurrence. Because presence/absence may under-represent fields that have large use (i.e. a single duck is equivalent to 1000s), we weighted the binomial likelihood function by the square root of the observed abundance plus 1.0 [38].

This study was an evaluation of the variable drawdown practice as a whole, thus we were most interested in how the treatments compared to each other and to the ND fields over time, as implemented by individual private landowners. Water depth is a significant driver of waterbird use [12,22–24], and this practice intentionally manipulates the timing of drawdown, and thus shallow water depths, but did not prescribe specific water depths at specific times. We wanted to know if shallow habitat was more valuable later in the survey period because the landscape was drying overall. Thus, our initial set of 14 models for each guild included all possible combinations of treatment, date, quadratic of date, cubic of date, and year. We then added combinations of water depth, the quadratic of water depth, and proportion of survey area saturated to the higher performing models to see if these improved model fit, resulting in 22 models. This also allowed us to separate the effect of water conditions (depth and saturation) from an overall treatment effect. We included quadratic and cubic forms of date because we suspected that the relationship of waterbird density and date (which is correlated with depth) may not be linear. We included water depth and the quadratic of water depth as previous studies have highlighted non-linear associations between some waterbird guilds and water depth [12,22]. We also included an interaction term between treatment and date to account for potential differences in the effect of date on bird use within treatments since habitat availability on the landscape is changing during the six-week study period [14,17]. We included the proportion of survey area saturated because we wanted to account for fields that were still wet and providing good habitat for shorebirds, even with 0 cm of measured water depth, which are far different from dry fields. We did not include highly correlated (r > 0.7) variables in the same models. We ranked models for each guild using Akaike’s Information Criterion (AIC) [39]. We assessed the fit of our negative binomial models by evaluating residual plots for evidence of autocorrelation or deviance from normality. We assessed the fit of our logistic regression models by estimating the area under the receiver operating curve. We also fit intercept-only models for inclusion in model comparisons. We drew inference from the most competitive (e.g., ΔAIC < 2) model(s) in the final model set and evaluated relative support for completive models based on Akaike weights; the probability that each model is the best given the set of models evaluated [39].

To assess the overall effect of the treatments, we compared the estimated mean densities (shorebirds and wading birds) or probability of use (dabbling ducks and geese and swans) and 95% confidence intervals (CI; estimated using the Wald method [40]) for each treatment from the best supported model based on AIC for each guild. We also sought to understand how the differences among treatments vary over the course of the study, so we plotted the predicted density or probability of use for each day from the best supported model based on AIC and included 95% CIs. For daily predictions based on the best supported model for each guild, which all included water depth and percent of survey area saturated, we allowed the water depth and percent of survey area saturated to vary based on the observed means for each treatment. We used our bi-weekly measurements of water depth and percent of survey area saturated to estimate the means for each day of the study in each treatment. If year was in the best supported model, we averaged the two years to create one estimate per waterbird guild.

## Results

**Fig 2:**
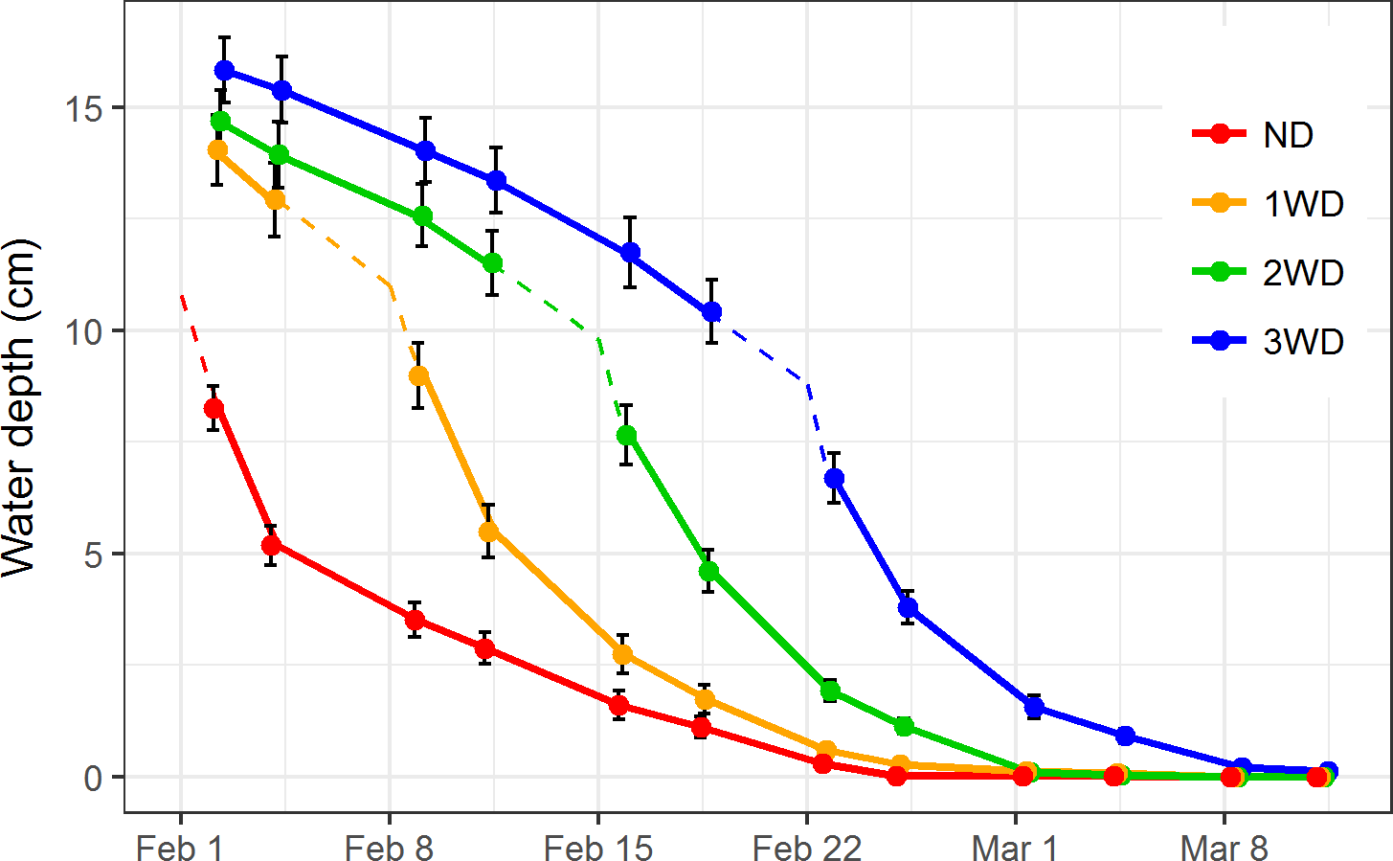
Mean water depth through time. Mean water depth (+/- standard error) for each survey occasion in each treatment over the six-week study period. The dashed portion of every line are our best estimates of water depth immediately prior to drawdown. The known start date of each drawdown treatment is indicated by the inflection in each dashed line. ND, no delay (red); 1WD, 1-week delay (orange); 2WD, 2-week delay (green); 3WD, 3-week delay (blue).

Water depths observed in drawdown treatments followed the predicted pattern (Fig 2). Depths slowly decreased due to seepage and evaporation until drawdown commenced, after which water depth decreased rapidly. Fields often remained puddled and saturated up to 2 weeks after the water was allowed to drain from the field. The ND fields drained slower than the treatment fields, which all drained at similar rates. The ND fields also began the study at lower water depths than the treatment fields, at likely the lowest depth allowable under the program (10 cm).

We observed 35 species of waterbirds among a total of 64,153 birds in 2012 and 62,282 birds in 2013 (Table 2). The three most numerous species were American coot (*Fulica americana*), northern pintail (*Anas acuta*), and dunlin (*Calidris alpina*), and the three most frequently encountered species were killdeer (*Charadrius vociferous*), ring-billed gull (*Larus delawarensis*), and greater yellowlegs (*Tringa melanoleuca*) (Table 2).

**Table 2:** Abundance and frequency of occurrence of waterbird guilds and associated species observed using rice fields implementing variable drawdown.

Common Name	Scientific Name	Treatment				Total	Freq. of occ. (% of surveys)
		ND	1WD	2WD	3WD		
**Geese and Swans**		**384**	**2718**	**4644**	**8913**	**16659**	**2.3**
Gr. White-fronted Goose	*Anser albifrons*	384	1407	2469	4084	8344	1.6
Snow Goose	*Chen caerulescens*		1255	1955	4538	7748	0.3
Ross's Goose	*Chen rossii*				221	221	0.0
Canada Goose	*Branta canadensis*				55	55	0.0
Tundra Swan	*Cygnus columbianus*		56	220	15	291	0.4
**Dabbling ducks**		**1537**	**4544**	**11543**	**17364**	**34988**	**12.9**
Gadwall	*Anas strepera*	2	5	1		8	0.1
Eurasian Wigeon	*Anas penelope*	1	2			3	0.1
American Wigeon	*Anas americana*	221	224	652	497	1594	0.8
Mallard	*Anas platyrhynchos*	155	278	373	1431	2237	3.2
Cinnamon Teal	*Anas cyanoptera*	2				2	0.0
Northern Shoveler	*Anas clypeata*	24	671	1568	3145	5408	3.0
Northern Pintail	*Anas acuta*	896	2618	6027	7525	17066	3.7
Green-winged Teal	*Anas crecca*	209	94	623	1981	2907	1.4
Unknown Dabbling Duck	*Anas* spp.	27	652	2299	2785	5763	0.7
Diving ducks							
Canvasback	*Aythya valisineria*			243	5	248	0.3
Ruddy Duck	*Oxyura jamaicensis*	1		28	1	30	0.1
**Wading birds**		**117**	**154**	**208**	**631**	**1110**	**12.3**
Great Blue Heron	*Ardea herodias*	29	20	32	58	139	2.2
Great Egret	*Ardea alba*	38	64	110	205	417	4.6
Snowy Egret	*Egretta thula*	24	12	54	46	136	0.9
Black-crowned Night- Heron	*Nycticorax nycticorax*	11				11	0.0
Sandhill Crane	*Antigone canadensis*	15	58	12	322	407	0.6
**Shorebirds**		**3756**	**7639**	**12588**	**13946**	**37929**	**40.6**
Black-bellied Plover	*Pluvialis squatarola*	31	49	187	108	375	0.9
Killdeer	*Charadrius vociferus*	1740	1907	3694	2448	9789	15.5
Greater Yellowlegs	*Tringa melanoleuca*	208	326	595	515	1644	6.8
Lesser Yellowlegs	*Tringa flavipes*		4	4	5	13	0.2
Long-billed Curlew	*Numenius americanus*	237	192	335	465	1229	5.9
Western Sandpiper	*Calidris mauri*		4	35	24	63	0.2
Least Sandpiper	*Calidris minutilla*	554	1128	2633	4570	8885	4.5
Unknown Peep	*Calidris mauri/minutilla*		23	53	200	276	0.1
Dunlin	*Calidris alpina*	556	3773	4525	5089	13943	3.2
Dowitcher spp.	*Limnodromus* spp.	55	23	88	21	187	0.2
Wilson's Snipe	*Gallinago delicata*	375	209	439	501	1524	3.2
Other waterbirds							
White-faced Ibis	*Plegadis chihi*	1610	1918	5406	2865	11799	3.8
American Coot	*Fulica americana*	1998	2688	5117	9640	19443	6.1
Ring-billed Gull	*Larus delawarensis*	1060	610	1124	1250	4044	13.5
California Gull	*Larus californicus*	1		5		6	0.1
Herring Gull	*Larus argentus*	13	20	125	21	179	0.6
	**Total abundance**	10477	20291	41031	54636	**126435**	
	**Species richness**	29	30	32	32	**35**	

Waterbird guilds in bold were included in data analysis. Note that not all treatments are sampled equally (Table 1). ND, no delay; 1WD, 1-week delay; 2WD, 2-week delay; 3WD, 3-week delay; Total, total counts over entire study; Freq. of occ., frequency of occurrence is the percent of surveys on which the species was detected.

As expected, we found significant variation in bird density through the six-week study period (30 January– 9 March 2012 and 4 February– 15 March 2013) and the effect of treatment varied over time for three of the four guilds evaluated. Based on AIC, all models for all guilds were an improvement over the intercept-only model (Table 3). For shorebirds, the most competitive model included the interaction of treatment and date, with date represented as a third order polynomial, year, the quadratic of water depth and the proportion of survey area saturated (Table 3). No other models were competitive (ΔAIC < 2). For wading birds, the best supported model included treatment, the third order polynomial for date, year, quadratic of water depth, and proportion of survey area saturated (Table 3). The model with an interaction between treatment and date received an AIC weight of 0.2 indicating there was some support for the interaction. For probability of use by dabbling ducks, the best supported model included treatment, the third order polynomial for date, year, quadratic of water depth, and proportion of survey area saturated. Similar to wading birds, the model with an interaction between treatment and date received some support with an AIC weight of 0.11. For geese and swans, two similar models shared almost equal weight. They included the interaction of treatment and date, with date represented as a second or third order polynomial, year, the quadratic of water depth and the proportion of survey area saturated. We opted to select the simpler of the two models (second order polynomial for date) for further analysis.

**Table 3:** Summary of model results predicting density (shorebirds and wading birds) and probability of use (dabbling ducks and geese and swans) by waterbirds of the variable drawdown practice.

	* *	Shorebirds		Wading birds		Dabbling ducks		Geese and swans	
Model^a^	*k*^b^	ΔAIC^c^	*w*_*i*_^d^	ΔAIC^c^	*w*_*i*_^d^	ΔAIC^c^	*w*_*i*_^d^	ΔAIC^c^	*w*_*i*_^d^
Intercept only	4	333.6	0.00	129.7	0.00	907.6	0.00	389.9	0.00
trt	7	317.1	0.00	114.2	0.00	802.4	0.00	368.5	0.00
trt + date	8	285.4	0.00	84.6	0.00	178.7	0.00	113.2	0.00
trt + date + year	9	277.8	0.00	82.0	0.00	178.5	0.00	110.3	0.00
trt + date^2^	9	277.0	0.00	43.0	0.00	177.4	0.00	64.9	0.00
trt + date^2^ + year	10	271.4	0.00	40.2	0.00	177.1	0.00	63.9	0.00
trt + date^3^	10	268.7	0.00	38.8	0.00	178.4	0.00	65.0	0.00
trt + date^3^ + year	11	264.1	0.00	36.5	0.00	177.6	0.00	63.7	0.00
trt × date	11	190.8	0.00	81.9	0.00	171.9	0.00	105.2	0.00
trt × date + year	12	179.4	0.00	79.6	0.00	172.5	0.00	103.0	0.00
trt × date^2^	12	169.6	0.00	35.9	0.00	169.8	0.00	36.2	0.00
trt × date^2^ + year	13	161.0	0.00	33.7	0.00	170.4	0.00	35.5	0.00
trt × date^3^	13	165.2	0.00	31.0	0.00	170.7	0.00	37.0	0.00
trt × date^3^ + year	14	156.6	0.00	29.2	0.00	171.0	0.00	36.1	0.00
trt × date^3^ + year + depth	15	117.9	0.00	25.8	0.00	68.8	0.00	27.1	0.00
trt × date^3^ + year + depth^2^	16	100.0	0.00	16.5	0.00	13.5	0.00	26.4	0.00
trt × date^3^ + year + depth^2^ + sat	17	**0.0**	**0.97**	2.5	0.21	4.3	0.11	**0.02**	**0.50**
trt × date^2^ + year + depth^2^ + sat	16	9.8	0.01	8.9	0.01	31.5	0.00	**0.00**	**0.50**
trt × date + year + depth^2^ + sat	15	7.8	0.02	20.2	0.00	29.6	0.00	41.5	0.00
trt + date + year + depth^2^ + sat	12	51.8	0.00	18.4	0.00	23.7	0.00	42.8	0.00
trt + date^2^ + year + depth^2^ + sat	13	49.9	0.00	6.2	0.03	25.7	0.00	14.6	0.00
trt + date^3^ + year + depth^2^ + sat	14	41.8	0.00	**0.0**	**0.75**	**0.0**	**0.89**	13.5	0.00

Summary of generalized linear mixed-effects models predicting density or probability of use for four guilds of waterbirds based on 22 candidate models. Model set included combinations of treatment (trt), date (aligned between years to the start date), and year. The most parsimonious models (ΔAIC < 2) are shown in bold for each guild.

^a^ all models include random effects for field and farm

^b^ *k* = number of parameters in each model

^c^ ΔAIC = difference in Akaike's Information Criterion

^d^ *w*_i_ = Akaike weight; × indicates an interaction between variables.

**Fig 3:**
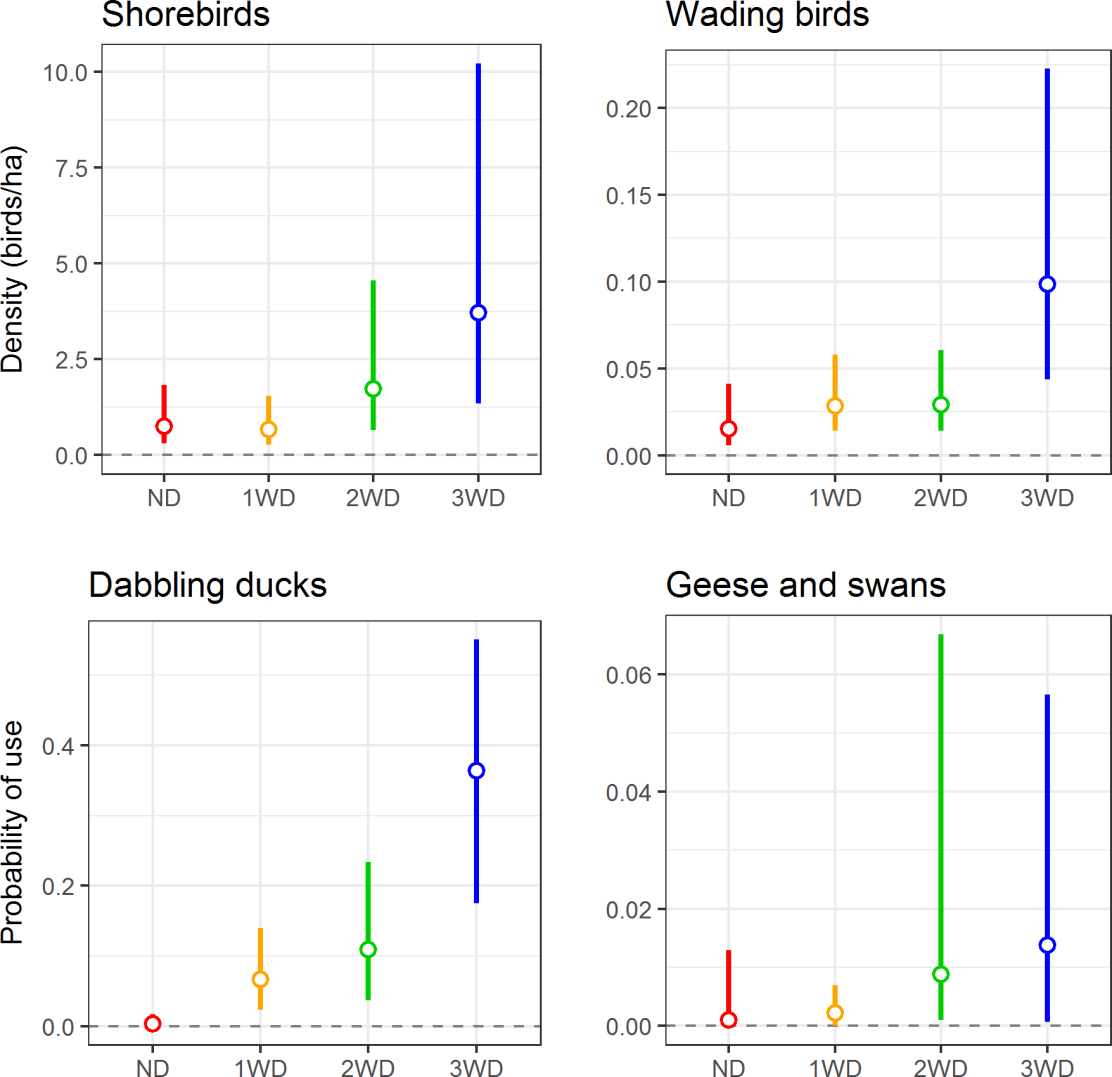
**Mean waterbird density (top) and probability of use (bottom) with 95% CIs estimated from best-supported models**. Mean waterbird density (top) or probability of use (bottom) with 95% confidence intervals for each variable drawdown treatment as estimated from the best-supported model, letting water depth and percent of survey area saturated vary as observed during the study. ND, no delay (red); 1WD, 1-week delay (orange); 2WD, 2-week delay (green); 3WD, 3-week delay (blue).

When we compared bird response to treatments overall, all four focal waterbird guilds showed similar patterns of higher use of later drawdown treatments (Table 2, Fig 3). The point estimates for mean density (shorebirds and wading birds) and probability of use (dabbling ducks and geese and swans) was highest in the 3WD treatment for all four guilds, and 2–3 times higher than the next highest treatment for shorebirds, wading birds and dabbling ducks. For shorebirds, the uncertainty around the 3WD estimate resulted in a small amount of overlap in 95% CIs between the 3WD and ND/1WD, resulting in no significant difference among treatments when considered from this conservative definition of significance. Mean density of wading birds was significantly higher in 3WD compared to ND. Probability of use by dabbling ducks was significantly higher for all delayed treatments compared to the ND, and 3WD was significantly higher than ND and 1WD. There was a great deal of uncertainty around the estimates for geese and swans resulting in no significant differences among treatments.

**Fig 4:**
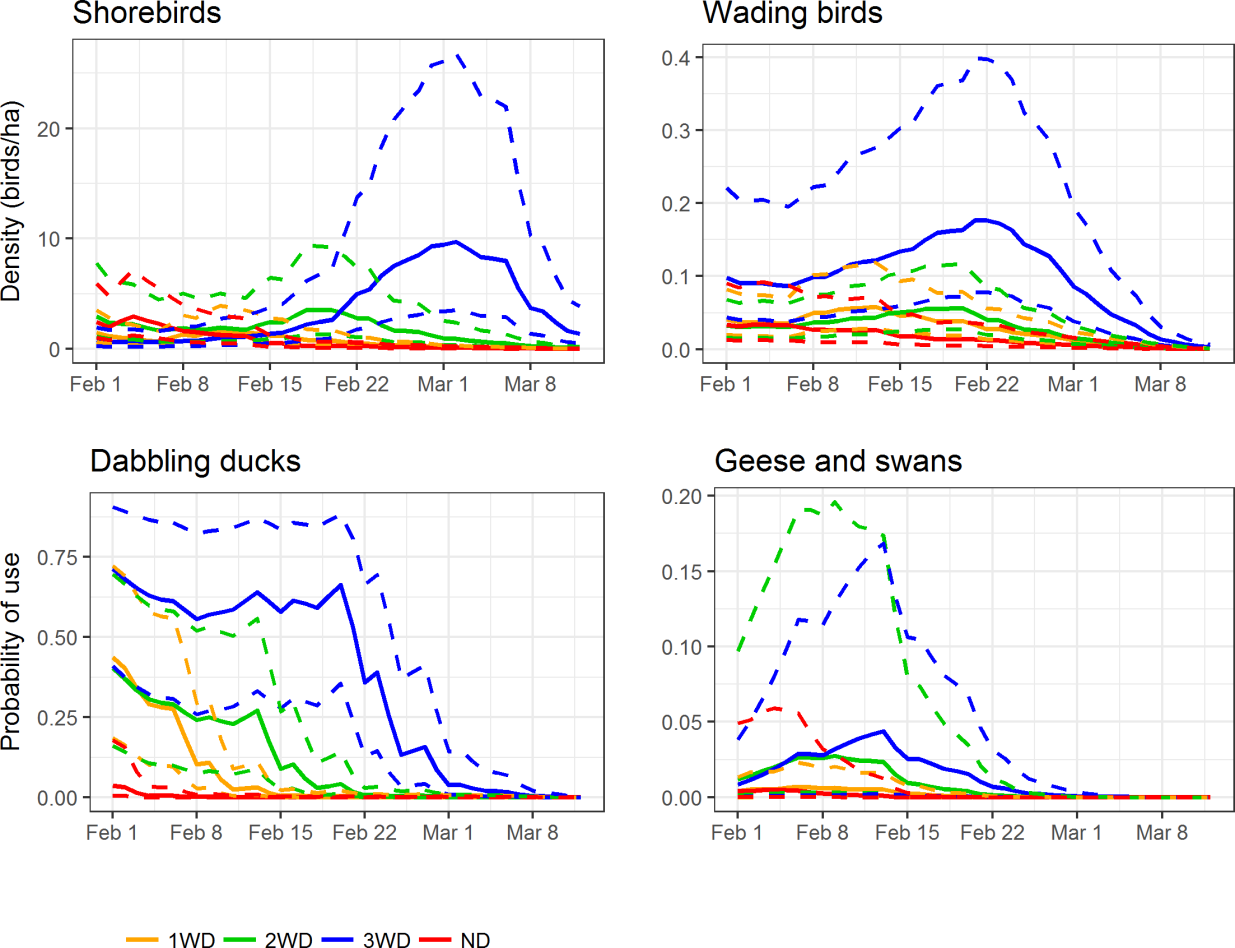
**Mean waterbird density (top) and probability of use (bottom) with 95% CIs over time as estimated by the top-ranked model**. Mean waterbird density or probability of use (solid lines) with 95% confidence intervals (dashed lines) in each variable drawdown treatment for each waterbird guild over the six-week study period as estimated by the best-supported models. ND, no delay (red); 1WD, 1-week delay (orange); 2WD, 2-week delay (green); 3WD, 3-week delay (blue).

When we plotted the bird responses over the course of the study, we found variation in the timing of higher use of treatments (Fig 4). While for shorebirds, the treatments did not differ significantly when averaged over the entire study period, the 3WD treatment had significantly higher estimated densities than the other treatments in March [peaking ~ March 1], and the densities in the 2WD peaked about 10 days earlier and were significantly higher than 1WD and ND for the last week in February. Similarly, when we predicted wading bird density over time, we found the 3WD treatment had significantly higher predicted densities than all other treatments for the second half of the study period. When we predicted probability of use by dabbling ducks over time, we found the 2WD to have significantly higher probability of use compared to ND and 1WD in mid-February, and the 3WD treatment had significantly higher probability of use compared to all other treatments for the last half of February. We found no significant differences in predicted goose and swan probability of use over time.

For all guilds, the top model included a quadratic effect of water depth as well as treatment and date and was an improvement over models that did not include one of these core variables. This indicated that there may be differences between treatments and date that are independent of water depth, however by design, treatment, depth and date were correlated.

## Discussion

We found that extending post-harvest flooding by three weeks past the traditional drawdown practice provided habitat for many waterbirds, including the four focal guilds we studied. Our results highlight the importance of maintaining flooding in rice fields beyond the traditional timeline and confirms the value of providing a diversity of water depths on the landscape to provide habitat for a diversity of waterbirds [11,23]. All treatments evaluated provided some habitat but the 3WD treatment was the best overall at providing habitat across guilds. The timing of highest density or probability of use of the 3WD was different across guilds and further highlights the benefit of this treatment to multiple guilds. Dabbing ducks and geese and swans had higher probabilities of use during the first half of the study, while wading birds and shorebirds reached peak densities in the middle and second half of the study. Every guild used every treatment and ND to some degree, illustrating that early drawdown treatments also have value for these guilds.

While all treatments provided some habitat to our focal guilds, the 3WD treatment was the best overall at providing habitat across guilds and across time. For shorebirds, the 3WD treatment is predicted to have higher densities in late-February and March than the other treatments even when they reached the same water depths. This provides evidence of shorebirds concentrating in remaining fields with suitable water depths due to the limited availability of shallow water on the landscape at that time [9,14,17]. During the timeframe of this study, the majority of shorebird species detected are wintering in the area and migration does not begin until later in March [7,16]. Thus the peak in shorebird density likely does not reflect an increase in shorebird densities across the landscape of the Sacramento Valley due to an influx of migrants. Predicted densities of wading birds was much greater in the 3WD treatment than the other treatments. A potential mechanism for this may be that when we looked closely at the species distribution within this guild we found Sandhill cranes (*Antigone canadensis*) used the 3WD treatment disproportionately more than the other treatments (five times more) and this species is known to roost in deeper water depths such as those available in the 3WD fields [41]. Dabbling ducks also had highest use in the 3WD treatment and we suspect this is due in part to the deeper water depths prevalent in the 3WD treatment. Geese and swans also had highest use in the 3WD treatment, although the 2WD treatment was not significantly different and their modeled probability of use was similar. For this guild, we cannot rule out migration away from the study area as a factor that may have influenced our results. Depending on the year, geese and swans may begin northbound movements during our study’s timeframe [9] and could be a factor in our low probabilities of use in the latter half of the study.

The 3WD treatment had the highest use by all four focal waterbird guilds but the reasons for that are different among guilds. Shorebirds use was greater due to the timing of the 3WD treatment, likely because it provided suitable shallow water depths at a time when shallow water was limited on the landscape. For dabbling ducks, geese and swans, and some wading birds, their higher use of the 3WD treatment was likely driven by the deeper depths available in 3WD, especially at the beginning of the study period.

Water depth (or presence of water at all) is an important driver of variation in bird use of rice fields, especially for shorebirds and dabbling ducks [12,22,42–44]. Water depth was dynamic in this study. Flooding was not actively maintained after the start of the study so water depths in the delayed drawdown rice fields declined over time due to seepage and evaporation. Then water depths changed quickly when drawdown was initiated, influencing the waterbird guilds using the fields (e.g. ducks leaving, shorebirds arriving). This practice successfully created a mosaic of water depths within each farm, creating suitable flooded habitat for a variety of species [25].

Mean densities in the initial (first) week of the study in all guilds and treatments were similar to densities found in other studies from the winter period in comparable post-harvest treatments [22,43,45]. Initial densities were overall much higher than those observed in a study in the same area from approximately 20 years earlier [12], however survey methods differed, some waterfowl populations have rebounded [6], and winter-flooding of rice had only just begun in the early to mid-1990’s. A recent study of a similar practice in the same study area, part of The Nature Conservancy’s BirdReturns program, reported densities similar to this study and reached even higher densities in April [46].

Recent energetic modeling studies found that the amount of flooded habitat is currently sufficient for the entire Central Valley to support the energy demands of waterbird populations during the February to mid-March timeframe, in years of normal precipitation [20,21]. However, our study found shorebirds concentrating in the delayed drawdown fields by the last week of February and it continued through the end of the study. While habitat availability across the larger Central Valley landscape may be sufficient for meeting shorebird population objectives until mid-March based on bioenergetic modeling, some individuals may need to fly farther to find food and they may find more competition for remaining resources. For example, research conducted in the study area found that dunlin used both rice and wetlands in February and then shifted to using wetlands more in March or left the study area [16] underscoring the complex interplay between rice and wetlands. Additionally, habitat and food resources provided by more flooded rice in late February and early March may reduce pressure on resources in managed wetlands and, theoretically, help resources last longer (K. Dybala per. comm.). Managed wetlands are typically flooded through March and the resources there could support shorebirds during migration in late-March and April when rice habitat is not available due to pre-planting activities. A similar case can be made for dabbling ducks, geese and swans, which were most abundant on 3WD fields in the first two weeks of the study. During times of drought, habitat deficits for waterfowl can occur as early as January [21] and practices such as variable drawdown may extend the resources available in wetlands for waterfowl.

Many factors can influence waterbirds’ selection of a field including the landscape context of a field, post-harvest practices, and food abundance. The abundance of suitable habitat (e.g. other flooded rice fields) and the distribution of key habitats (e.g. managed wetlands) in the surrounding landscape can heavily influence waterbird use of a particular field [32,33,47–50]. We controlled for this in our study design and analysis by having all the treatments clustered on each farm. The distribution of habitat in the surrounding landscape was represented similarly in all treatments on each farm and thus across the study as a whole so we do not believe our assessment is biased. By including a random effect of farm in our analysis, we controlled for the varying landscape contexts for each individual farm. The post-harvest practices applied to fields (e.g. flooding, disking, etc.) also influence waterbird use [12,43,51,52]. However, the degree to which these practices influence use during drawdown after 3 or more months of flooding is unknown. We observed that most fields during drawdown were mudflats with little topography and straw residue. Most of the differences in post-harvest practices occurred among farms, so again, the random effect of farm controlled for this. Post-harvest practices did not vary among treatments thus did not influence our comparisons.

Available food resources can effect waterbirds’ use of a field and many factors affect seed, vegetation, and prey abundance in rice fields such as post-harvest practices, pesticide and nutrient levels leftover from the growing season, water sources, and placement of the field on the landscape [51,53,54]. The clustered design of our fields and the random effect of farm in our analysis controlled for differences in food availability due to farm-level differences in the above factors prior to the start of variable drawdown. Once variable drawdown started, differences in food availability due to changes in flooding condition and/or time of year can be attributed to the treatment itself. The treatment we are evaluating is the timing of drawdown, and that timing and exposure drives food availability—whether food becomes available due to lowering water depths and the ability of some guilds to access new foods, or changes in invertebrate populations through the late-winter. As a consequence, food availability is confounded with treatment and we ultimately were unable to assess other possible mechanisms driving differences in bird abundance among treatments outside of water depth. For dabbling ducks and geese who forage on seeds, it is possible that the four weeks that pass between the first drawdown and the last drawdown result in less seeds available in the later drawdown due to increased decomposition and predation, for whatever seed resources are still available after three months of flooding [51,53]. That could mean that the effect of later drawdowns on waterfowl density could be even stronger if we could account for the loss of seeds over the 3–4 weeks of the study. For shorebirds, it is possible the opposite could be true, particularly given that when we controlled for depth, the 3WD was still significantly better than the ND or 1WD. It is unknown how benthic invertebrate communities mature over the course of the winter in rice fields but we know zooplankton populations can be quite high [55]. It is possible that the three weeks that pass between the first drawdown and the last drawdown (Feb 1 to Feb 21) result in some increase in invertebrate densities since days are getting longer (but air temperatures are not necessarily getting warmer). Either way, changes in food availability would essentially be a result of the treatments themselves.

Waterbird use of rice changes over the diel cycle depending on many factors. It has been documented throughout the world that dabbling ducks, and geese and swans, often forage in rice at night and in greater numbers than during the day [51,56,57], although day time use of rice fields in California is common [12,22,45]. Shorebirds, too, have been found to use forage and even roost in rice fields at night [16]. While some waterbird densities may be higher at night than during the day, all surveys of all treatments were completed during the day so this should not result in any bias in the comparisons among treatments within this study.

As with any study involving private landowners, there was variation in their ability to adhere to the starting depth requirements in the practice, which was exacerbated by the drought conditions unfolding at the time (2013–2015). In both years of the study, participating rice growers received all the water they requested to initially flood the fields after harvest in November. For most growers their last opportunity to add additional water is mid-January as many do not have access to water deliveries later in winter. Most growers did add more water to the fields to ensure they could meet minimum starting depths. However, early 2012 was very dry and one grower in our study that relied partially on rainfall to maintain water depths was unable to meet the requested starting depth of 10 cm in all their fields. This resulted in not having enough water in the last two treatments (2WD and 3WD) when it came time to draw them down. 2013 was still early in the drought and all of the growers were again allocated sufficient water for the study, but the same low starting depths occurred at a few farms. ND fields began the study with average depths lower than treatment fields. This is likely attributable to some participating farms not wanting to pay for water to top off a field they are going to drain right away, coupled with needing to overfill the treatment fields to ensure there is water to draw down in the fields later in February. Additionally, some growers chose their slowest draining field for the ND field, which likely caused the shallower decline in water depths compared to the other treatments. The use of farm as a random effect in models accounted for some of this farm-specific variation, as did the inclusion of water depth as a variable. This study took place in the first two years this practice was offered, and there was a learning curve for both landowners and project administrators on how to ensure optimal depths are achieved.

The post-harvest flooding of rice fields has become an important component of waterbird conservation in the Central Valley. The practice of variable drawdown as described in this study has been a part of U. S. Department of Agriculture’s Natural Resources Conservation Service conservation incentive programs since 2011. Thus far, up to 20% of California’s rice acreage was enrolled in this practice annually, extending habitat availability for waterbirds. Programs that share the cost of practice implementation and support innovative strategies for maintaining habitat are important to supporting waterbirds in the Pacific Flyway. We found the highest densities of shorebirds in the 3WD treatment during the last three weeks of the study, and recent models identified March and April as habitat-limited for shorebirds [20]. Furthermore, another habitat creation program in the same geography, BirdReturns, observed a robust response by waterbirds to shallow-flooded rice fields maintained later in March and April [46]. Thus we suggest delaying the beginning of the variable drawdown process for two weeks or more, extending the availability of shallow flooded habitat later into March or even April. However, we recognize there is a balance to be met. Later drawdowns are riskier for rice growers—potentially limiting participation and the habitat a program can provide. Delayed drawdown coupled with a significant rain event could delay planting activities and jeopardize a grower’s livelihood. Later drawdowns are also hindered by the availability of surface water to flood fields in February and March; many water vendors cease deliveries in January to perform annual maintenance on canals.

## Conclusions

Staggering the drawdown of winter-flooded rice fields, as this study has shown, could add value to any rice fields that are flooded for rice residue decomposition and/or hunting opportunities in other parts of the world [58] and could potentially be added to agri-environmental schemes. Our study found that delaying the drawdown of rice fields by three weeks from the traditional timing supported the highest response by waterbirds. This practice successfully created a mosaic of water depths which provided habitat for a diversity of waterbird guilds. Finding innovative ways of providing flooded habitat on agricultural land is especially important in highly-modified landscapes where agriculture complements wetland habitat and is an important component of waterbird conservation. Variable drawdown proved useful in rice, and a similar staggering of water drawdown could also be used in other annual crops, such as corn (*Zea mays*) and wheat (*Triticum aestivum*), that can be flooded seasonally for wildlife, in California and in other parts of the world.
